# Comprehensive surgical treatment for obstructive rectal endometriosis: a case report and review of the literature

**DOI:** 10.1186/s12905-022-01858-z

**Published:** 2022-07-07

**Authors:** Yumei Xu, Yixin Xu, Lu Miao, Meng Cao, Wei Xu, Linsen Shi

**Affiliations:** 1 Department of Radiation Oncology Center, The Affiliated Hospital of Xuzhou Medical University, 99 West Huaihai Road, Xuzhou, 221006 Jiangsu People’s Republic of China; 2 Department of Gastrointestinal Surgery, The Affiliated Hospital of Xuzhou Medical University, 99 West Huaihai Road, Xuzhou, 221006 Jiangsu People’s Republic of China; 3 Department of Obstetrics and Gynecology, The Affiliated Hospital of Xuzhou Medical University, 99 West Huaihai Road, Xuzhou, 221006 People’s Republic of China

**Keywords:** Rectal endometriosis, Obstruction, Comprehensive surgical treatment

## Abstract

**Background:**

Intestinal obstruction caused by endometriosis maybe easily misdiagnosed as a tumor or other occupying disease in emergency condition. How to deal with it depending on the clarity of the preoperative diagnosis and the experience of the surgeon.

**Case presentation:**

A 47-year-old woman, admitted to our emergency service with abdominal pain and distension for 5 days, anal stop exhausting and defecating for 3 days. Based on imaging and laboratory examination, we made a preoperative diagnosis of rectal endometriosis probably. After 7 days of colon decompression with a intestinal obstruction catheter, an operation of laparoscopic partial rectal and sigmoid resection without protective stoma and total hysterectomy was performed successfully. The patient obtained a smooth postoperative course and doing well after 12-weeks follow up.

**Conclusions:**

Obstruction caused by rectal endometriosis is very rare and easily overlooked by surgeon and gynecologist. Appropriate preoperative diagnosis and preoperative management can reduce the trauma and incidence of complications.

## Background

Endometriosis, defined as functional endometrial tissues growing in other areas outside the uterine cavity and accompanied by debilitating chronic pelvic pain, is estimated to affect about 6–10% of reproductive women [1]. Rectal endometriosis is one of the uncommon sub-types of deep infiltrating endometriosis that may cause severe clinical symptoms, such as pain, bleeding and obstruction [2–4].

Treatment of endometriosis is an enormous challenge for clinicians because it is difficult to cure and easy to relapse [5]. Hormonal suppressive therapy may be applied to reproductive women with mild symptoms [6]. In most cases, surgical resection is an efficient option for rectal endometriosis especially with obstruction [7]. But in an emergency condition, how to grasp the best “window of opportunity” for the operation still depending on the clarity of the preoperative diagnosis and the experience of the surgeon.

Herein, we report a case of rectal obstruction caused by endometriosis in a middle-aged woman, who was dealt with interdisciplinary comprehensive means.

## Case presentation

**Fig. 1:**
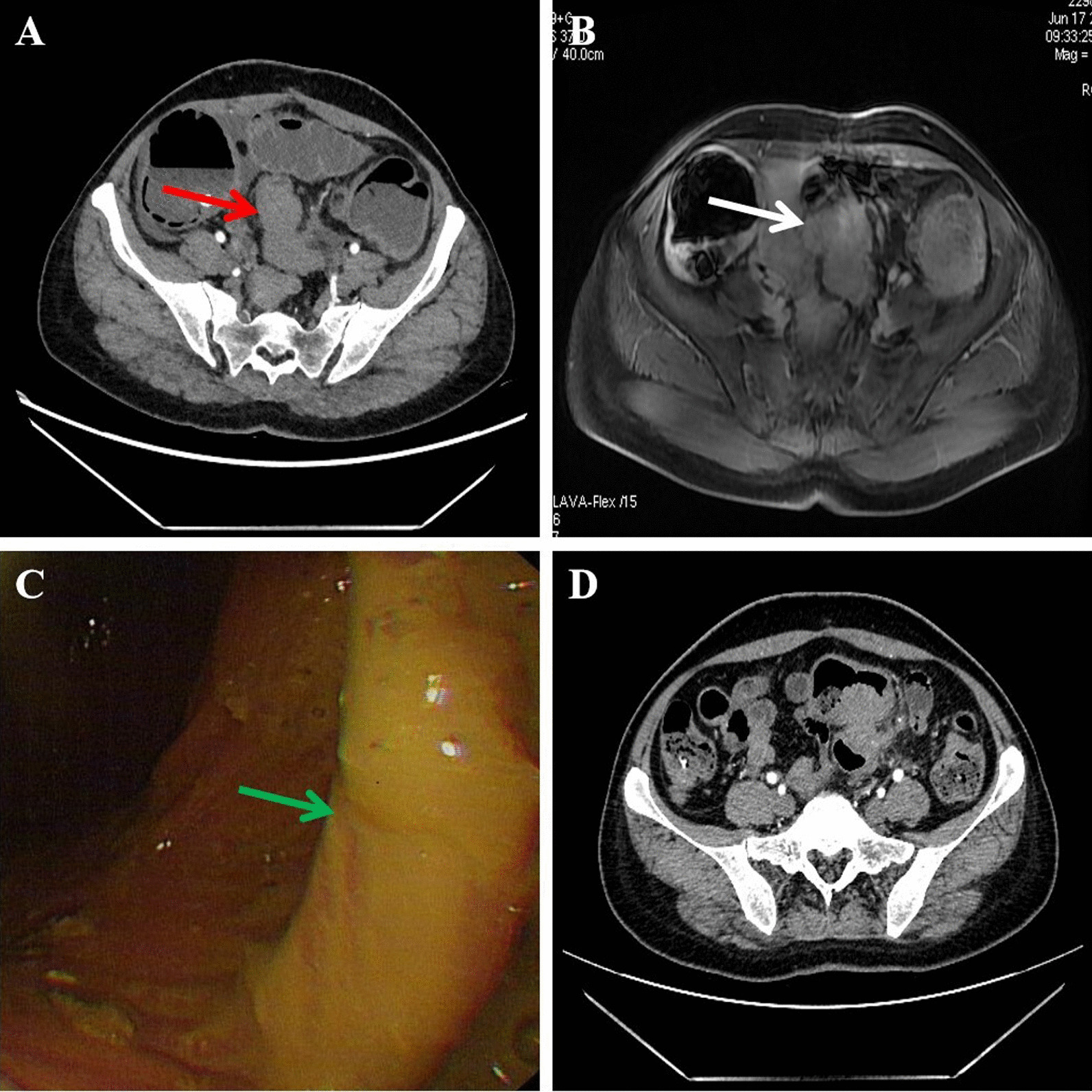
Preoperative examination of the patient. **A** Computed tomography (CT) revealed wall thickening from the upper rectum to sigmoid, accompanied by low intestinal obstruction (red arrow). **B** Magnetic resonance imaging (MRI) revealed neoplastic lesions of the sigmoid colon (white arrow). **C** Colonoscopy showed stenosing tumor formation in the upper rectum, the mucosal surface is smooth (green arrow). **D** After 7 days of colon decompression, computed tomography reexamination showed the dilatation of the colon was significantly less than before

A 47-year-old woman, gravid 3 para 1, visited our hospital with complaints of abdominal pain and distension for 5 days. She was suffered with constipation accompanied by defecation for more than 1 year, menstrual exacerbation, no blood stool and fever, the menstrual cycle was also stably. She had a history of cesarean section 25 years ago and hysteromyoma excision 20 years ago. Physical examination only show left lower abdomen tenderness without peritoneal irritation. Digital rectal examination revealed no abnormalities. Her cancer antigen 125 level was 60.62 U/ml. Abdominal computed tomography (CT) revealed wall thickening from the upper rectum to sigmoid, secondary low intestinal obstruction and multiple myoma in the uterus (Fig. 1A). Colonoscopy showed a stenosing tumor formation in the upper rectum, but the mucous membrane is smooth (Fig. 1B). Biopsies only found nonspecific inflammation. Magnetic resonance imaging (MRI) also revealed neoplastic lesions of the sigmoid and rectum (Fig. 1C).

**Fig. 2:**
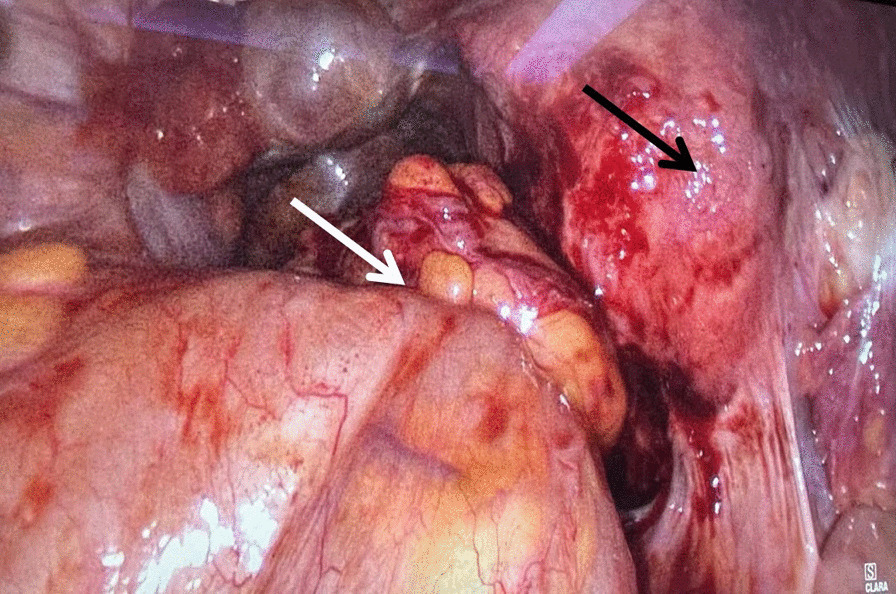
During the operation, a stenosis recto-sigmoid mass (white arrow) and multiple adenomyoma of the uterus (black arrow) (H&E × 100)

A primary diagnosis of intestinal obstruction secondary to rectal endometriosis was made according to the laboratory and instrument examinations. After 7 days of colon decompression and nutrition support therapy, the patient's abdominal distension was significantly relieved, CT reexamination showed that the dilatation of the colon was significantly less than before (Fig. 1D). An operation of laparoscopic partial rectal and sigmoid colon resection and total hysterectomy was performed successfully. The specimen was removed through a small abdominal incision (about 5 cm). During the operation, we found a stenosis recto-sigmoid mass and multiple adenomyoma of the uterus. The mass had adhesion with the posterior lamina of right broad ligament and the posterior uterine wall, without infiltration into the rectovaginal septum (Fig. 2).

**Fig. 3:**
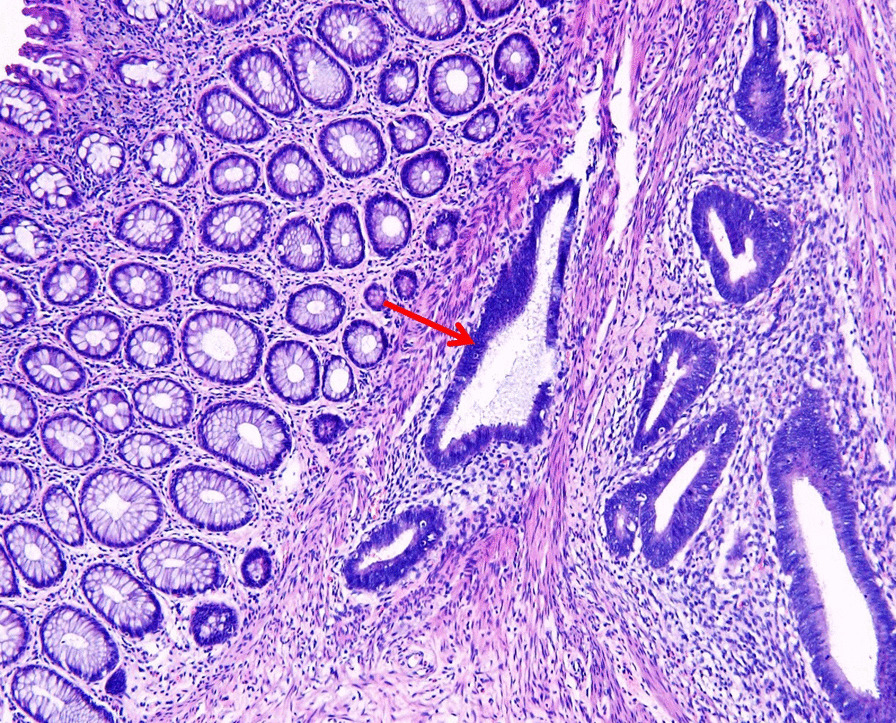
Sections from the rectum specimen showed endometrioid structures between the rectal muscle walls (red arrow)

Postoperative pathology showed endometrioid structures between the rectal muscle walls, chronic inflammation of the remaining mucous membranes, multiple endometriosis of the uterus and ovaries, confirmed the diagnosis of rectal endometriosis (Fig. 3). The patient discharged 10 days post operation. After 12 weeks of follow-up, the patient did not complain of special discomfort and recovered well.

## Discussion and conclusions

Endometriosis is one of the most common benign diseases in gynecology defined as the presence of endometrial tissue or active foci outside the uterine cavity and accompanied by chronic inflammation [8]. So far, the etiopathogenesis of endometriosis have not been fully elucidated. “Retrograde menstruation” is one of the most widespread theories, but several recent studies indicate that bowel permeability, transformation of microbiome and metabolic profile might also play a role in the pathogenesis of this chronic disease [9–11]. According to different lesion sites, endometriosis can be broadly characterized into four sub-types: Ovarian endometriosis, peritoneal, deep infiltrating endometriosis (DIE) and endometriosis of other locations [12–14]. The prevalence of DIE is rare and only account for 1–2% of patients suffered with endometriosis [15].

Even though ultrasonographic combined with histopathological examination is the gold standard for the diagnosis of endometriosis, it is very difficult to obtain enough positive tissue samples before surgery with DIE [16]. Therefore, an adequate collection of medical history, specific laboratory tests, additional imaging and endoscopy techniques may be helpful in the initial diagnosis before operation [5]. Multiple imaging modalities have been applied to preoperative diagnose of DIE, transvaginal pelvic sonography (TVS) has been reported with a sensitivity and specificity of 71–98% and 92–100% respectively [17]. However, MRI signs can also be valuable in the preoperative diagnosis of rectal DIE and accurately predict the need for segmental resection [18].

Although ovarian and peritoneal endometriosis represent the majority of endometriotic implants within the pelvis, DIE is the most difficult and challenging type in treatment [19]. For the asymptomatic rectal DIE an medical management may be utilized [20], but when accompanying obstruction, surgery is required at most of time. Surgical methods can be divided into three types: disc resection, shaving excision and segmental resection. Until now, there is no consensus on the choice of specific surgical procedures for rectosigmoid endometriosis (RSE), The exact mode of surgical procedure depends on the location, size, number of lesions, and extent of bowel constriction, as well as the experience and expertise of the surgeon [21, 22]. Two retrospective cohort studies have confirmed that conservative surgery is preferred to radical surgery in patients with RSE because of preserving intestinal neurological activity and associated with less short-term complications [23, 24]. As obstructive rectal DIE was often admitted first by a gastrointestinal (GI) surgeon, a multidisciplinary collaboration include but not limited to GI surgeon, gynecologic surgeon, pathologist and radiologist should be raised during the perioperative period.

The techniques of laparoscopic nerve-sparing firstly reported by Ceccaroni may result in lower rates of postoperative sexual, rectal and urinary dysfunctions than classical approaches and [25]. But according to the research of Spagnolo et al., they found that bowel as well as urinary dysfunction in patients with a bulky posterior endometriotic nodule often present before surgery, a nerve-sparing surgery just not provoke any de novo intestinal and urinary dysfunction or worsen the preexisting dysfunction [26]. As a benign lesion, radical resection is not indispensable, and attention should be paid to the preservation of pelvic autonomic nerve function during the operation, minimize the impact on pelvic anatomy and postoperative quality of life [27].

This patient was diagnosed with intestinal obstruction secondary to rectal endometriosis after all the laboratory and instrument examinations were completed. If emergency surgery is performed, a prophylactic ileostomy may be required during the operation due to the intestinal edema and risk of postoperative anastomotic fistula. According to the multidisciplinary discussion, an intestinal obstruction catheter was placed through anus with colonoscopy, nutritional support therapy and intestinal cleanup were also performed. So, for this patient, only segmental bowel resection was performed while preserving the pelvic plexus and mesenteric vascular as reported previously [28], the quality of life was almost unaffected post operation.

In summary, complete obstruction caused by rectal endometriosis is rare and require the attentions of GI surgeon. Accurate preoperative diagnosis and adequate preoperative preparation can reduce the trauma and incidence of complications. A multidisciplinary collaboration is recommended at most of situation.

## Data Availability

All data generated during this study are included in this published article.

## Abbreviations

CT: Computed tomography
MRI: Magnetic resonance imaging
TVS: Transvaginal pelvic sonography
DIE: Deep infiltrating endometriosis
RSE: Rectosigmoid endometriosis
GI: Gastrointestinal
